# Prevalence of Underdiagnosed Fragile X Syndrome in 2 Health Systems

**DOI:** 10.1001/jamanetworkopen.2021.41516

**Published:** 2021-12-30

**Authors:** Arezoo Movaghar, David Page, Murray Brilliant, Marsha Mailick

**Affiliations:** 1 Waisman Center, University of Wisconsin−Madison, Madison, Wisconsin; 2 Department of Biostatistics and Bioinformatics, Duke University, Durham, North Carolina

## Abstract

This cross-sectional study examines the gap between best estimates of prevalence and clinical diagnosis of fragile X syndrome by mining the electronic health records of 3.8 million people in Wisconsin.

## Introduction

Fragile X syndrome (FXS) is the most common inherited cause of intellectual disability and autism with substantial challenges for patients and their families.^1,2,3^ Diagnosing FXS is challenging because of its clinical heterogeneity, absence of evident physical characteristics at birth, variation of phenotypes between the sexes, and similarity of phenotypes with other conditions.^1,2,3,4^ It is recommended that all individuals with developmental delay, intellectual disability, and/or autism of an unknown cause should be genetically tested for FXS.^5^ However, only a small fraction of individuals receive referral for testing.^6^ It is widely believed that FXS is substantially underdiagnosed in the general population. This study aims to quantify the gap between best estimates of prevalence and clinical diagnosis by mining the electronic health records (EHRs) from 3.8 million people.

## Methods

This cross-sectional study was approved by the institutional review boards at the University of Wisconsin–Madison and Marshfield Clinic Research Institute. Only deidentified EHR data was used for this study and therefore informed consent was waived. We followed the Strengthening the Reporting of Observational Studies in Epidemiology (STROBE) reporting guideline.

We obtained the deidentified EHRs from 2 separate comprehensive health care systems in Wisconsin. The digitized Marshfield Clinic Health System included approximately 40 years (1979-2018) of medical records for 1.7 million patients. The University of Wisconsin Health System included 2.1 million patients, with approximately 33 years (1988-2021) of digitized health data. Race and ethnicity data were self-reported, but these data were not available for all participants.

We identified all participants who received the diagnostic code for FXS (using *International Classification of Diseases, Ninth Revision [ICD-9] *code 759.83 and *International Statistical Classification of Diseases and Related Health Problems, Tenth Revision [ICD-10] *code Q99.2) on at least 2 occasions to eliminate those who were tested for FXS without further evidence of a positive clinical diagnosis. We used the most recent meta-analysis of prevalence based on published reports of population-based screening carried out by Hunter et al^4^ to calculate the expected number of cases. Hunter’s review includes a large number of published population-based studies and represents the lowest bound for the expected number of cases (1.4 per 10 000 in males and 0.9 per 10 000 in females).^4^ The threshold for statistical significance was *P* < .05, and testing was 1-sided. Statistical analysis was performed using R version 3.6.3 (R Project for Statistical Computing) from April to September 2021.

## Results

The longitudinal EHRs of 3 807 512 patients were examined; 1 984 369 patients (52.1%) were female; ages in the EHR system included the full range from 0 years to 89 years and older. The expected number of cases was estimated to be 435 individuals (256 male individuals, 179 female individuals). However, only 142 (104 male individuals, 38 female individuals) were clinically diagnosed (Table 1). Among those diagnosed with FXS who had data on race and ethnicity in their EHR, 84.6% were self-reported as White. The population proportion analysis (see eAppendix in the Supplement) showed that there was a significant difference between the number of individuals who actually received the diagnosis and the prevalence estimates (Table 2). The rate of clinical diagnosis in the Marshfield Clinic population was estimated to be 28.06%, meaning that 71.94% of individuals with FXS did not receive proper clinical diagnosis. A similar rate of diagnosis was observed in the UW Health population (36.40%). The estimated rate of underdiagnosis in women was considerably higher than in men (86.75% vs 61.06% in Marshfield Clinic; 71.88% vs 58.04% in UW Health).

**Table 1.** Characteristics of Individuals Clinically Diagnosed With FXS^a^

	Median (range)					
	UW Health			Marshfield Clinic		
	All cases (n = 87)	Male (n = 60)	Female (n = 27)	All cases (n = 55)	Male (n = 44)	Female (n = 11)
Age at the time of data extraction, y	30.0 (4.0-84.0)	29.5 (4.0-84.0)	31.0 (4.0-76.0)	29.0 (9.0-93.0)	29.5 (9.0-93.0)	25.0 (18.0-61.0)
Age at FXS diagnosis, y	13.0 (<1.0-84.0)	10.5 (<1.0-84.0)	17.0 (1.0-64.0)	13.0 (<1.0-92.0)	15.0 (<1.0-92.0)	11.0 (1.0-51.0)

Abbreviation: FXS, fragile X syndrome

^a^ No significant differences were observed between the 2 populations in terms of age at the time of data extraction and age of FXS diagnosis.

**Table 2.** FXS Diagnosis Rate

	Patients, No.			Estimated rate of underdiagnosis, %	*P* value, population proportion
	Total	Clinically diagnosed FXS cases	Expected FXS cases		
**UW Health**					
All	2 084 289	87	239	63.60	<.001
Male	1 018 259	60	143	58.04	<.001
Female	1 063 894	27	96	71.88	<.001
**Marshfield Clinic**					
All	1 723 223	55	196	71.94	<.001
Male	802 832	44	113	61.06	<.001
Female	920 385	11	83	86.75	<.001

Abbreviation: FXS, fragile X syndrome.

## Discussion

By investigating population-based medical data, we found a high rate of FXS underdiagnosis in the general population. Similarity of diagnosis rate in Marshfield Clinic and UW Health suggests that the rate of underdiagnosis is not reflective of practices of specific health care systems and instead shows the complexity of the diagnostic process. The milder level of symptoms, due to having the second (unaffected) X chromosome, contributes to the higher rate of underdiagnosis among female individuals. The substantial gap between population prevalence and clinical practice indicates the urgent need to identify barriers to receiving a clinical diagnosis. Developing more effective screening practices (ie, newborn, universal premutation, and smart prescreening) may accelerate the diagnostic process and facilitate patients’ access to timely intervention and services.

A limitation of this study was that both populations were relatively homogenous with 84.6% of those diagnosed with FXS self-reporting as White. Therefore, additional studies on more diverse populations are required. The statistical methods used for this study did not allow us to calculate indicators of uncertainty for our estimates of underdiagnosis.

## Notes

Supplement. **eAppendix.** Proportion Population
