# Supplementary material for: Prevalence of Underdiagnosed Fragile X Syndrome in 2 Health Systems
Source: JAMA Netw Open. 2021 Dec 30;4(12):e2141516. doi: 10.1001/jamanetworkopen.2021.41516 (PMC8719235; doi:10.1001/jamanetworkopen.2021.41516)
Supplement: Supplement. — eAppendix. Proportion Population [file jamanetwopen-e2141516-s001.pdf]

Supplemental Online Content

Movaghar A, Page D, Brilliant M, Mailick M. Prevalence of underdiagnosed fragile X syndrome in 2 health systems. *JAMA Netw Open.* 2021;4(12):e2141516. doi:10.1001/jamanetworkopen.2021.41516

eAppendix. Proportion Population

This supplemental material has been provided by the authors to give readers additional information about their work.

© 2021 Movaghar A et al. *JAMA Network Open.*

eAppendix. Population Proportion

We used one-tailed population proportion test where the null hypothesis was that *the proportion of individuals clinically diagnosed with FXS (p') is equal to estimated prevalence of the disease (p).*

The alternative hypothesis was that *"the proportion of individuals clinically diagnosed with FXS (p') is lower than estimated prevalence of the disease (p)."*

To calculate the Z score we used the following formula:

$$Z = \frac{p' - p}{\sqrt{\frac{p(1-p)}{n}}}$$

Where n is total number of patients. If p-value is less than 0.05 we have significant evidence to reject the null hypothesis and accept the alternative. R version 3.6.3 was used to perform the statistical analysis for this study.

© 2021 Movaghar A et al. *JAMA Network Open.*
